# The prognostic value of the NPT test combined with amplitude integrated electroencephalogram in children with VE and its bioreliability analysis

**DOI:** 10.5937/jomb0-43317

**Published:** 2024-01-25

**Authors:** Yinghong Liu, Wenjuan Li, Chaohai Wang, Shuyun Chen, Gaiqing Wang

**Affiliations:** 1 Children's Hospital Affiliated to Shanxi Medical University, Pediatric Intense Care Unit, Taiyuan, China; 2 Shanxi Cancer Hospital, Intensive Care Unit, Taiyuan, China; 3 Children's Hospital of Shanxi Province (Shanxi Maternal and Child Health Care Hospital), Pediatric Intense Care Unit, Taiyuan, China; 4 Children's Hospital of Shanxi Province (Shanxi Maternal and Child Health Care Hospital), Department of Neurology, Taiyuan, China; 5 Shanxi Medical University, Department of Neurology, Taiyuan, China; 6 Hainan Medical University, Sanya Central Hospital (Hainan Third People's Hospital), Department of Neurology, Sanya, China

**Keywords:** shengxin analysis, viral encephalitis, the new poison, amplitude integrated EEG, shengkin analiza, virusni encefalitis, novi otrov, amplitudni integrisani EEG

## Abstract

**Background:**

Viral encephalitis (VE) is one of the common diseases of children with intracranial infection, it has come on urgent, progress is fast, and the clinical features of severe cases may even lead to disability, death, and other serious adverse prognostic outcomes, so seek in early diagnosis and prognosis of efficiency of the relevant indicators to stop in time and take effective means to prevent the further development is of great significance. Neopterin (NPT), as a factor that plays an important role in the process of validation development, has been relatively rarely studied in children with VE.

**Methods:**

In this study, 127 cases of children with VE were retrieved from the TCGA database by bioinformatics, and their amplitude integrated electroencephalogram (AEEG) related information was collected at the same time. The neurodevelopmental status of VE children was evaluated according to the Gesell scale and divided into the good group (n=88) and the poor group (n=39). The differences in NPT expression and AEEG score between them were observed. In addition, the clinical data of 100 children without VE were screened from the database, and the differences in NPT expression and AEEG score between VE children and non-VE children were compared. The ROC curve was used to evaluate the clinical efficacy of NPT combined with AEEG in diagnosis and prognosis prediction. Kaplan-Meier was used to observe the effect of NPT high expression and low expression on poor prognosis of VE children.

## Abstract (sr)

**Uvod:**

Virusni encefalitis (VE) je jedna od čestih bolesti dece sa intrakranijalnom infekcijom, hitno je napredovala, napreduje brzo, a kliničke karakteristike teških slučajeva mogu čak dovesti do invaliditeta, smrti i drugih ozbiljnih prognostičkih ishoda, pa je traženje u ranoj dijagnostici i prognozi efikasnosti relevantnih indikatora da se na vreme zaustavi i preduzme efikasna sredstva za sprečavanje daljeg razvoja od velikog značaja. Neopterin (NPT), kao faktor koji igra važnu ulogu u procesu razvoja validacije, relativno je retko proučavan kod dece sa VE.

**Metode:**

U ovoj studiji, 127 slučajeva dece sa VE je preuzeto iz baze podataka TCGA bioinformatikom, a istovremeno su prikupljene informacije vezane za njihov amplitudski integrisani elektroencefalogram (AEEG). Neurorazvojni status dece sa VE procenjen je prema Gezelovoj skali i podeljen na grupu dobar (n=88) i lošu grupu (n=39). Uočene su razlike u ekspresiji NPT i AEEG skoru između njih. Pored toga, klinički podaci 100 dece bez VE su pregledani iz baze podataka i upoređene su razlike u ekspresiji NPT i AEEG skoru između VE dece i dece bez VE. ROC kriva je korišćena za procenu kliničke efikasnosti NPT u kombinaciji sa AEEG u dijagnozi i predviđanju prognoze. Kaplan-Meier je korišćen za posmatranje efekta visoke i niske ekspresije NPT na lošu prognozu dece sa VE.

**Rezultati:**

U poređenju sa decom koja nisu VE, deca VE su imala više NPT i niže AEEG rezultate, dok je siromašna grupa imala veći NPT i niži AEEG skor. ROC kriva je pokazala da je površina ispod krive NPT u kombinaciji sa AEEG-om za dijagnozu i predviđanje prognoze VE bila 0,892 i 0,884, respektivno, što je više od onih kod pojedinačnog NPT-a ili AEEG-a, što ukazuje da kombinovana detekcija ima visoku kliničku sliku, vrednost ne samo za dijagnozu već i za predviđanje i prognozu. Kaplan-Meierove krive preživljavanja pokazale su da visoka ekspresija NPT često predviđa lošu prognozu.

**Zaključak:**

Kombinacija NPT i AEEG moze se koristiti za blagovremenu i efikasnu procenu stanja dece sa VE u procesu kliničke prakse.

## Introduction

Meningitis refers to diffuse inflammatory changes in the pia mater, most of which are caused by infection with pathogenic microorganisms, including bacteria, viruses, fungi, etc. [1]. As one of the common types of meningitis, viral meningitis is an acute inflammatory disease of the meninges caused by viral infection. The clinical manifestations are mainly fever, headache, and meningeal irritation. Some meningitis caused by viruses transmitted by the respiratory tract is infectious [2] [3]. The incidence of viral meningitis is high in summer and autumn and can occur in all age groups, but the incidence is high in children, especially in those less than 1 month old and with weak immune function [4]. The early symptoms of viral meningitis in infants are not typical, Mainly characterized by fever, rash, vomiting, and poor mental state, but as the disease progresses, easy to delay the best treatment time, maybe on children with brain or nerve function damage, leading to poor prognosis, therefore, early recognition and diagnosis and reasonable treatment is the key to improve the prognosis of children with viral meningitis [5]. Currently, VE diagnosis is primarily based on clinical symptoms, cerebrospinal fluid analysis, and neuroimaging. However, these methods have limitations in their sensitivity and specificity. Therefore, there is a need to identify new biomarkers that can improve VE diagnosis and prognosis.

NPT is one of the intermediates in the biosynthesis pathway of guanosine triphosphate and tetrahydrobiopterin in vivo, and it is also a coenzyme in the hydroxylation process of phenylalanine, tyrosine, and tryptophan. It is a basic substance with low relative molecular weight and is synthesized from GTP by GTP-cyclic hydrolase I [6]. At present, neopterin has been detected only in humans and primates, and its biological stability is good. At present, the research on neopterin has been involved in various diseases related to the activation of the cellular immune response, including infection, trauma, tumor, autoimmunity, and other inflammatory diseases [7]. It is also used to predict the course of disease and assess the prognosis, such as malignant diseases and infections caused by human immunodeficiency virus (HIV). It is also helpful to assist in the diagnosis and differentiation of diseases with or without cellular immune involvement and can be used as a predictor of cardiac events in patients with acute coronary syndrome (ACS) [8]. All the above statements indicate that NPT plays an important role in a variety of inflammatory diseases, but the relationship between NPT and children with VE remains unclear. Therefore, this study used bioinformatics methods to retrieve relevant information about NPT in children with VE from TCGA and analyze its impact on the prognosis of children with VE. In addition, AEEG, as a simplified form of EEG newly developed this year, is of great significance for the assessment of the degree of early brain function damage [9]. Amplitude integrated electroencephalogram (AEEG) is a non-invasive technique that can provide valuable information on the neurodevelopmental status of children with VE. Combining these two methods may have high clinical value for VE diagnosis and prognosis prediction. Therefore, this study further combined NPT with AEEG and observed the predictive efficacy of the combined detection on the diagnosis and prognosis of VE children.

## Materials and methods

### Data sources

Bioinformatics was used to retrieve and download the clinical data of VE children with NPT in TCGA, and the corresponding cases were followed up until June 2022. In this study, we retrieved 127 cases of children with VE from the TCGA database by bioinformatics. We included children who met the following criteria: (1) diagnosed with VE based on clinical symptoms, cerebrospinal fluid analysis, and neuroimaging; (2) aged between 1 month and 18 years at the time of diagnosis; (3) with AEEG-related information available in the database; and (4) with complete clinical data and follow-up information. We excluded children who had a history of other neurological or psychiatric disorders, or who had received treatments that might affect the NPT or AEEG measurements.

We also selected 100 children without VE from the same database as the control group. These children were matched with the VE group in terms of age, sex, and follow-up duration.

The study was approved by the Institutional Review Board of Shanxi Medical University, and the data used in this study were obtained from the TCGA database, which is publicly available and de-identified. Therefore, the Institutional Review Board waived the need for informed consent from the parents or guardians.

### Methods

### AEEG examination and evaluation method

AEEG was monitored by a Japanese electrooptic EEG-1200C central monitoring system. According to the international 10–20 system, scalp electrodes were placed using 11 leads (mid frontal point (Fz), central point (Cz), vertex (Pz), left frontal pole (Fp1), right frontal pole (Fp2), left frontal (F3), right frontal (F4), left middle temporal (T3), right middle temporal (T4), left occipital (O1), right occipital (O2)). Bilateral ear electrodes (A1, A2) were used for reference to record brain electrical activity, synchronously monitor left and right eye movements, mandibular electromyography, heart rate, respiration, transcutaneous oxygen saturation, and other physiological signals, which are used to judge sleep cycle and identify false errors. Routine monitoring for 3~4 hours, at least 1~2 waking-sleep cycles, can be extended or shortened according to the needs of the child’s condition. Parameters such as continuity, sleep cycle, the amplitude of the lower boundary, the width of the pop band, and amplitude change of the lower boundary in AEEG graphics were evaluated according to the method of reference [10]. A double-blind evaluation was performed by two physicians with high AEEG experience. The results of AEEG graphics were scored according to the scoring system, and the quality control was performed by physicians from the neuro electrophysiology center. The total score ranged from 0 to 13, with lower scores indicating a higher degree of AEEG abnormalities.

### Gesell scale

The scale mainly evaluates the neurodevelopmental ability of infants from four aspects, including motor ability, response-ability, speech ability, and response-ability. The development quotient is calculated according to the relationship between the score of the scale and the age of the child. A development quotient 75% is considered normal neurodevelopment, and 75% is considered abnormal neurodevelopment. According to the evaluation results, VE children were divided into a good neurodevelopment group and a poor neurodevelopment group.

### NPT measurement

The NPT levels were measured by two independent observers blinded to the patient’s clinical data. The inter-rater reliability and intra-rater reliability of the NPT test were evaluated using the intraclass correlation coefficient (ICC). For inter-rater reliability, the NPT levels of 20 randomly selected children were measured by two observers, and the ICC was calculated. For intra-rater reliability, one observer measured the NPT levels of the same 20 children twice, with a one-week interval between measurements, and the ICC was calculated.

### aEEG recordings

The aEEG recordings were performed by trained technicians blinded to the patient’s clinical data. The inter-rater reliability and intra-rater reliability of the aEEG recordings were evaluated using Cohen’s kappa coefficient. For inter-rater reliability, the aEEG recordings of 20 randomly selected children were scored by two observers, and Cohen’s kappa coefficient was calculated. For intra-rater reliability, one observer scored the aEEG recordings of the same 20 children twice, with a one-week interval between scorings, and the Cohen’s kappa coefficient was calculated.

### Statistical analysis

All data in the study were collected and put into Statistical Product and Service Solutions (SPSS) 25.0 statistical software (IBM, Armonk, NY, USA) for data analysis. (1) Measurement data: A normality test was performed on the data first. If the data obeyed normal distribution and homogeneity of variance, it was expressed as (⎯x±s). Paired sample T was used for testing within the group, and variance was compared between the groups. (2) Count data: The percentage was used for descriptive statistical analysis, and the X2 test was used. (3) Survival analysis: Kaplan-Meiersurvival curve was drawn for the prognosis and survival rate of patients in the two groups. (4) ROC evaluation of NPT combined with AEEG in the diagnosis and prognosis of VE children. P < 0.05 indicates a significant difference.

## Results

The differences in NPT and AEEG scores between VE children and non-VE children were analyzed.

### Differences in baseline data

The baseline data showed that there was no significant difference in age and gender between the two groups, indicating that there was no bias in the research results caused by uncontrollable factors. See Table 1 for details.

**Table 1:** Comparison of baseline data.

Group	Number	Age<br>(month)	Gender	
			Man	Woman
VE children	127	4.11±1.49	67 (52.76)	60 (47.24)
non-VE<br>children	100	4.10±1.36	54 (54.00)	46 (46.00)
t/x^2^		0.053	0.035	
P		0.958	0.852	

### The NPT differences

**Figure 1:**
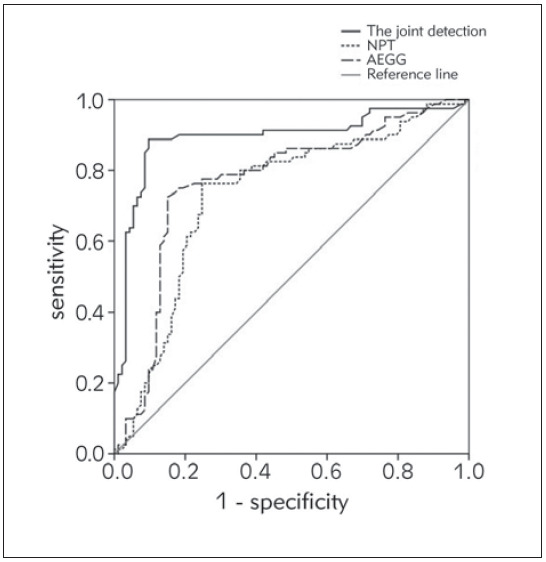
Correlation analysis of visfatin with severity of AMI. (A) Correlation between visfatin and NT-proBNP. (B) Correlation between visfatin and CTnI. (C) Correlation between visfatin and MYO. (D) Correlation between visfatin and CK-MBm. The content of visfatin in the peripheral blood was positively correlated with NT-proBNP, CTnI, MYO and CK-MBm in AMI patients (P<0.01).

Figure 1 shows the differences in NPT between the two groups in detail. Among them, the NPT level of VE children was (26.44±6.71) nmol/L, and that of non-VE children was (13.17±2.79) nmol/L, which showed significant differences.

### The difference in AEEG score

Table 2 shows the differences in AEEG total score and each dimension score between the two groups. It can be seen that the total score and each dimension score of the children with VE are significantly lower than those of the children without VE, indicating that they have significant abnormal performance in AEEG performance.

**Table 2:** AEEG score was different between the two groups.

Group	n	Score of<br>continuity	Sleep cycle	Lower boundary<br>amplitude	Variation of pop band<br>width and lower<br>boundary amplitude	Total
VE children	127	1.28±0.65	3.04±0.93	1.46±0.75	2.88±0.75	8.66±1.85
Non-VE children	100	2.04±0.85	4.00±0.83	2.44±0.50	3.96±0.72	12.44±1.07
t		-7.665	-8.106	-11.156	-10.904	-18.125
P		<0.001	<0.001	<0.001	<0.001	<0.001

### Diagnostic efficacy of NPT combined with AEEG for VE

**Figure 2:**
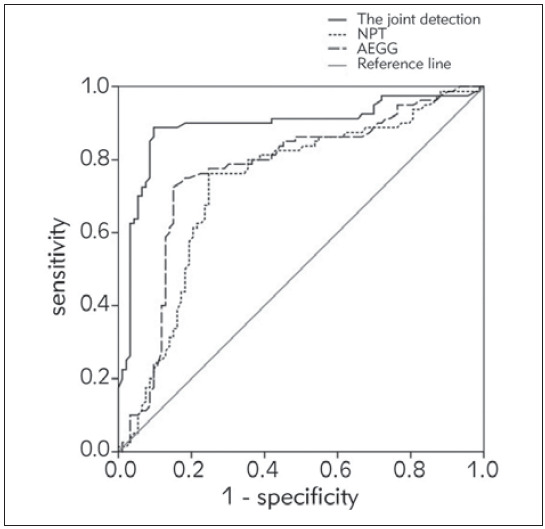
ROC of NPT combined with AEEG on VE.

The diagnostic efficiency of each index is shown in Table 3. Figure 2 shows the ROC curve, which shows that NPT combined with AEEG has a high predictive efficiency for the early diagnosis of VE, and the area under the curve is significantly higher than that of NPT and AEEG alone.

**Table 3:** Diagnostic performance table.

	95%CI	Sensitivity (%)	Specificity (%)	AUC	Cutoff value
The joint detection	0.836~0.947	88.30	87.30	0.892	—
NPT	0.653~0.809	72.10	78.30	0.731	19.29 nmol/L
AEEG	0.693~0.843	73.40	65.40	0.768	10.12 point

### The differences in NPT and AEEG scores between the good group and the poor group of VE children were analyzed

### Differences in baseline data

The main basis for the division of the good and poor groups of VE children was the Gesell scale score. Table 4 shows that the Gesell score of the good group was significantly higher than that of the poor group, but there was no significant difference in age and gender between the two groups.

**Table 4:** Comparison of baseline data.

Group	Number	Gesell score	Age<br>(month)	Gender	
				Man	Woman
Good group	88	70.26±3.49	4.03±1.50	48 (54.55)	40 (45.45)
Bad group	39	57.75±3.09	4.28±1.49	19 (48.72)	20 (51.28)
t/x^2^		19.277	-0.863	0.368	
P		<0.001	0.390	0.544	

### The NPT differences

**Figure 3:**
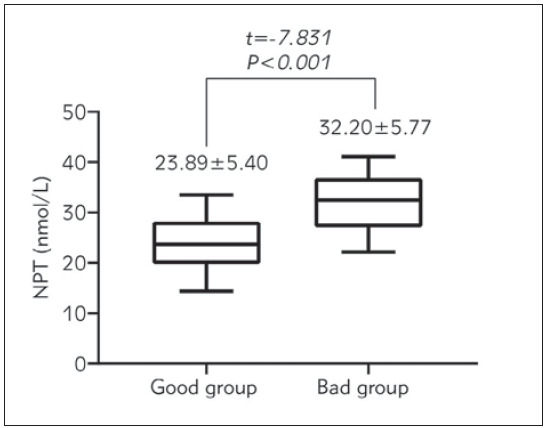
NPT difference between good group and poor group.

Figure 3 shows the differences in NPT between the poor group and the good group in detail. The NPT level of the good group was (23.89±5.40) nmol/L, and that of the poor group was (32.20± 5.77) nmol/L, showing a significant difference between the two groups.

### AEEG differences

Table 5 shows in detail the differences in AEEG scores and scores of each dimension between the good group and the poor group of VE children. It can be seen from the table that the total AEEG scores and scores of each dimension of the two groups were significantly lower than those of the good group except for the lower boundary amplitude score, and although there was no significant difference in the lower boundary amplitude score between the two groups, you can see that the lower boundary amplitude in the bad group is on average slightly lower than that in the good group.

**Table 5:** AEEG difference between good group and Bad group.

Group	n	Score of continuity	Sleep cycle	Lower boundary<br>amplitude	Variation of pop<br>band width and	Total
Good group	88	1.43±0.50	3.50±0.50	1.49±0.50	3.02±0.80	9.44±1.17
Bad group	39	0.92±0.81	2.00±0.83	1.41±1.14	2.56±0.50	6.90±1.90
t		4.343	12.583	0.539	3.292	9.224
P		<0.001	<0.001	0.591	0.001	<0.001

### Efficacy of NPT combined with AEEG in evaluating the prognosis of children with VE

**Figure 4:**
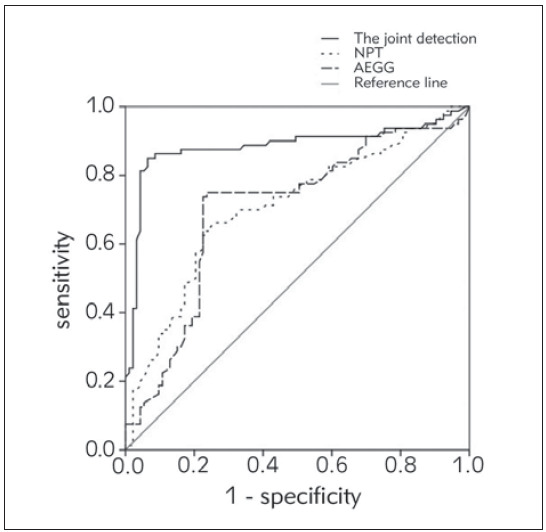
NPT combined with AEEG was used to evaluate the prognosis ROC of VE children.

The diagnostic efficacy of each index is shown in Table VI. Figure 4 shows the ROC curve of each index. It can be seen that the area under the curve of NPT combined with AEEG is significantly higher than that of any single test, indicating that NPT combined with AEEG has an important evaluation value in predicting the prognosis of children with VE.

**Table 6:** Diagnostic performance table.

	95%CI	Sensitivity (%)	Specificity (%)	AUC	Cutoff value
The joint detection	0.824~0.943	81.50	90.30	0.884	—
NPT	0.628~0.786	76.30	66.40	0.707	27.39 nmol/L
AEEG	0.622~0.783	67.40	67.20	0.702	8.39 point

### The effect of NPT on the prognosis of VE children

**Figure 5:**
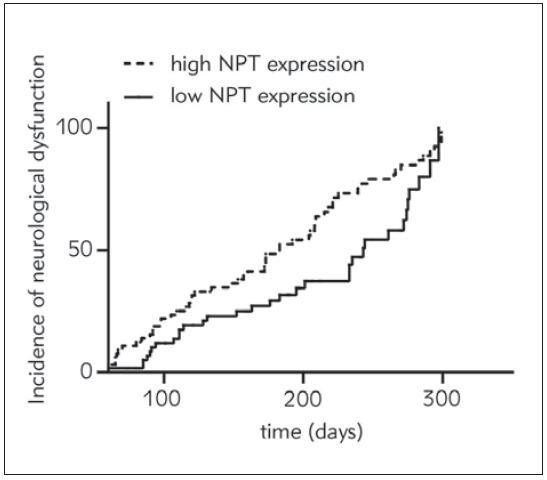
Kaplan-Meier survival curve was used to observe the effect of NPT expression on the prognosis of VE children.

With the mean NPT of VE children as the dividing line, the children above the mean were included in the NPT high expression group, and the children below the mean were included in the NPT low expression group. The follow-up time was up to June 2022. The Kaplan-Meier survival curve was drawn to observe the effect of NPT expression on the prognosis of VE children. The results showed that the number of children with neurological dysfunction in the NPT high expression group was significantly higher than that in the NPT low expression group, indicating that the higher the NPT expression, the worse the prognosis of VE children, as shown in Figure 5.

## Discussion

NPT, which is derived from CTP and synthesized and released by monocytes/macrophages activated by Y-interferon (IFN-Y) secreted by T lymphocytes, is a sensitive signal of cell-mediated immune activity [11]. Some scholars pointed out that in a variety of infectious diseases, especially viral infections) in blood and urine NPT levels, high levels of the NPT and systemic inflammatory disease, sepsis, and surgical stress reaction between closely related, such as measurement of the human body fluids, including blood, cerebrospinal fluid, the NPT, will help to further observation of the cellular immune response [12]. In inflammatory and autoimmune diseases of the central nervous system (CNS), the production of NPT in cerebrospinal fluid is produced by immune cells and neurons under the stimulation of IFN-Y. As for the changes in the level of NPT in cerebrospinal fluid during CNS inflammatory diseases, recent studies have reported that the NPT in cerebrospinal fluid is increased in acute encephalitis and other CNS acute inflammatory diseases [13]. Related studies have reported that the NPT level of cerebrospinal fluid is significantly increased during herpes virus encephalitis, suggesting that the intracranial immune activity is obvious at this time. It is also believed that the increased production of NPT in cerebrospinal fluid can be used as a marker of intracranial immune activation. NPT in cerebrospinal fluid is a useful and widely used inflammatory marker for acute or chronic CNS diseases. Therefore, NPT may be used as a predictive indicator for intracranial inflammatory infectious diseases [14].

The results of this study showed that the NPT level of children with VE was significantly higher than that of children without VE, and in the population of children with VE, the NPT of the poor group was significantly higher than that of the good group, which indicated a trend. The more severe the degree of VE, the higher the expression level of serum NPT, which could also be shown by the results of this study 4. Kaplan-Meier survival curve showed that the prognosis of children with high NPT expression was significantly worse than that of children with low NPT expression. It is suggested that cell-mediated immune activity in the cerebrospinal fluid of the body is significantly enhanced during intracranial infection, especially in viral encephalitis [15].

In addition, AEEG can visually display the signals of the double-top cerebral cortex through electroencephalogram and monitor brain function in real-time. The device is simple to operate and not easy to be interfered with by other factors, which has high diagnostic value for brain injury [16]. The results of this study showed that the AEEG scores of VE children were significantly lower than those of non-VE children, and the expression of AEEG scores was lower in the poor group. First of all, in terms of EEG continuity, When the fertilized egg gradually split the proliferation and differentiation, form a complete, fetal brain development also gradually improves, during this period, these are the central junction formation and migration of neurons proliferation, dendritic axon growth, form synaptic pruning start and myelination organization neural network formation, electroencephalogram (EEG) also started by discrete state into a discrete/continuous alternating graphics state, Finally, continuous EEG is formed, so when the continuity score is poor, it can indicate abnormal neural development of children [17]. In addition, from the perspective of the sleep cycle, the sleep cycle can effectively reflect the development of the brain. The EEG in the quiet sleep period shows a discontinuous background wave and broad spectral band, while the active sleep period is a narrow spectral band and continuous background wave. With the gradual maturation and completeness of brain development, the EEG in the sleep cycle phase gradually changes to the natural, regular, and mature cycle state. However, due to viral infection of the brain parenchyma, VE children cannot maintain a natural and regular sleep cycle, thus showing a decrease in sleep cycle score [18]. In addition, the reduced amplitude of the upper and lower boundaries and the width of the wave in the EEG indicate abnormal brain development, which is consistent with the results of this study. The AEEG scores of VE children with poor prognoses are significantly lower [19].

Since NPT and AEEG have specific manifestations in children with VE, the ROC of this study was drawn to evaluate the diagnostic and prognostic efficacy of NPT combined with AEEG for VE. According to the results, the area under the curve of NPT combined with AEEG for the diagnosis and prognostic prediction of VE were 0.892 and 0.884, respectively. It is significantly higher than that detected by NPT or AEEG alone, indicating that the combination of NPT and AEEG can be used to timely and effectively evaluate the condition of children with VE in the process of clinical practice, and corresponding measures can be taken to prevent the further development of the disease, which is of great significance to improve the prognosis of children with VE [20] [21].

## Dodatak

### Data Availability

The simulation experiment data used to support the findings of this study are available from the corresponding author upon request.

### Acknowledgments

None.

### Conflict of interest statement

All the authors declare that they have no conflict of interest in this work.
